# Versatile Effects of GABA Oolong Tea on Improvements in Diastolic Blood Pressure, Alpha Brain Waves, and Quality of Life

**DOI:** 10.3390/foods12224101

**Published:** 2023-11-12

**Authors:** Chih-Cheng Lin, Chih-Yu Hsieh, Li-Fen Chen, Yen-Chun Chen, Tien-Hwa Ho, Shao-Chin Chang, Jia-Feng Chang

**Affiliations:** 1 Department of Biotechnology and Pharmaceutical Technology, Yuanpei University of Medical Technology, Hsinchu City 300, Taiwan; lcc@mail.ypu.edu.tw; 2 Department of Food Science, Yuanpei University of Medical Technology, Hsinchu City 300, Taiwan; cyuh0817@gmail.com (C.-Y.H.); davidchenyenchun@gmail.com (Y.-C.C.); 3 Institute of Tea & Pottery Culture, Yuanpei University of Medical Technology, Hsinchu City 300, Taiwan; rita520912@gmail.com; 4 Department of Pet Healthcare, Yuanpei University of Medical Technology, Hsinchu City 300, Taiwan; 5 Department of Information Management, Yuanpei University of Medical Technology, Hsinchu City 300, Taiwan; howa@mail.ypu.edu.tw; 6 Department of Physical Science and Technology, Yichun University, Yichun 336000, China; william@alchemytech.com.tw; 7 Division of Nephrology, Department of Internal Medicine, Taoyuan Branch of Taipei Veterans General Hospital, Taoyuan City 330, Taiwan; 8 Department of Nursing, Yuanpei University of Medical Technology, Hsinchu City 300, Taiwan; 9 School of Medicine, National Yang-Ming University, Taipei City 120, Taiwan; 10 Renal Care Joint Foundation, New Taipei City 220, Taiwan

**Keywords:** γ-aminobutyric acid, oolong tea, blood pressure, brain wave, quality of life

## Abstract

Emerging evidence has demonstrated that using a new manufacturing technology to produce γ-aminobutyric acid (GABA)-fortified oolong (GO) tea could relieve human stress and exert versatile physiological benefits. The purpose of this human study was to investigate the therapeutic effects of daily GO tea consumption on improvements in blood pressure, relaxation-related brain waves, and quality of life (QOL) over a period of 28 consecutive days. Total polyphenols, major catechins, and free amino acids were analyzed via an HPLC assay. Changes in heart rate, blood pressure, α brain waves (index of relaxation), and the eight-item QOL score were investigated on days 0, 7, 14, 21, and 28. The chemical analysis results showed that GO tea contained the most abundant amino acids and GABA, contributing to the relaxation activity. Among all study participants, the daily consumption of GO tea could reduce systolic blood pressure on day 21 and diastolic blood pressure on day 28 (*p* < 0.05 for both). For participants with pre-hypertension, GO tea could effectively reduce heart rate and systolic and diastolic blood pressure on day 28 (*p* < 0.05). At the end of the study, incremental changes in alpha brain waves and QOL scores were also demonstrated (*p* < 0.05 for both). This study suggests that GO tea might potentially serve as a natural source for alternative therapy to improve blood pressure, stress relief, and QOL.

## 1. Introduction

The maintenance of autonomic nervous system (ANS) homeostasis is prone to be disturbed due to stress and the comorbidities intricately associated with generalized anxiety disorder and long-term depression [1]. Mental stress can alter the autonomic control of the heart and evoke an increase in heart rate (HR) and blood pressure [2]. In light of this, a pathophysiological link may exist between psychology and hypertension. Indeed, evidence indicates that anxiety is intricately associated with depression, hypertension, and ANS imbalance [3]. Moreover, anxiety plays a more pivotal role than depression in the development of hypertension [3].

γ-Aminobutyric acid (GABA), a ubiquitous non-protein amino acid, is an inhibitory neurotransmitter that improves insomnia, depression, and anxiety and thereby reduces stress-induced blood pressure elevation [3,4,5]. The underlying physiological roles of GABA are associated with the modulation of neurohumoral transmission, the attenuation of sympathetic activation, incremental changes in alpha waves, and the prevention of neurological disorders [3,4,5]. Our prior research found that mackerel fish protein hydrolysate containing various amino acids and GABA could mitigate fatigue and blood pressure and improve the quality of life (QOL) of the study participants [6]. Nonetheless, information regarding the therapeutic effects of GABA on mental stress in humans remains scarce. Among the four types of brain waves (alpha, beta, delta, and theta) used for mental stress measurements with the electroencephalogram, alpha brain wave activation resulting from wakeful relaxation serves as an index of anxiety relief [7]. Under stress conditions, GABA administration not only reduces anxiety but also enhances immune modulation [8]. Accordingly, it is of great importance to investigate the versatile roles of GABA in foods in mood stabilization, stress relief, autonomic balance and anti-hypertensive effects to benefit human health and QOL.

Teas containing abundant polyphenols and amino acids are proven to have many health-beneficial bioactivities. Among various types of tea, semi-fermented oolong tea is a popular beverage consumed in Taiwan. The therapeutic applications of teas to promote feelings of calmness, decrease excitation, and relieve stress are considered to correlate with their contents of amino acids generated by prolonged anaerobic fermentation, especially L-theanine and arginine [9,10]. Herein, we developed a new type of GO tea through the synergistic use of anaerobic fermentation to increase GABA content in the oolong tea extract (above 150 mg/100 g). Compared with common oolong tea, GABA-fortified oolong (GO) tea was found to exhibit greater bioactive effects on relaxation [11]. In people with acute stress, GO tea effectively alleviated autonomic imbalance, as determined by measuring heart rate variability [11]. To the best of our knowledge, GABA studies concerning mental stress relief determined by directly measuring brain wave changes are lacking. Therefore, we devised a brand-new idea that the daily consumption of GO tea could improve alpha brain waves and benefit hypertension and QOL. Collectively, the aim of this study was to compare bioactive components (polyphenols, catechins, and amino acids) and influences on the heart rate, blood pressure, alpha brain waves, and QOL between famous Taiwanese teas and GO tea in human participants.

## 2. Materials and Methods

### 2.1. Teabag Preparation and Comparison of pH Values between Various Teas

Various tea products were obtained from the local market for teabag preparation, including Wenshen Paochong (WP) tea (New Taipei, Taiwan), High-mountain Oolong (HO) tea (Chiayi, Taiwan), Oriental Beauty (OB) tea (Hsinchu, Taiwan), and Tongding Oolong (TO) tea (Nantou, Taiwan). GO tea containing more than 150 mg/100 g GABA was derived from Sijichun tea (Nantou, Taiwan). The above tea products were used to make tea bags (4 g in each tea bag). All study participants consumed two tea bags per day and brewed the tea by pouring 200 mL of boiling water onto each bag. GABA tea was manufactured through enzymatic decarboxylation from glutamic acid under anaerobic conditions [12]. Given that the biosynthesis of GABA is tightly correlated with the pH level in the environment, the pH values of the various teas were also compared.

### 2.2. Extraction and Measurement of Phytochemicals

The amounts of total phenolics, catechins, and free amino acids in the various tea extracts were determined by high-performance liquid chromatography (HPLC) and colorimetric assay, as reported previously [13]. The total contents of phenolic compounds and catechins in teas were then determined by using a standard curve prepared with gallic acid and expressed in terms of milligrams of gallic acid equivalents (GAE) per gram of extract solid. The free amino acid contents were measured according to previous research [14] and expressed as milligrams of theanine equivalents (TE) per gram of dry weight.

### 2.3. Human Study

Online public advertisements were posted on social media to recruit research participants. Adults aged between 40 and 85 years who were not pregnant, had no record of brain surgery, and were not taking any medications that affect the nervous system fulfilled our inclusion criteria. Exclusion criteria included left-handedness; a history of medical or neurological diseases, psychiatric disorders, or head trauma; the consumption of central-nervous-system-active drugs in the two weeks prior to study entry; or the presence of electroencephalographic abnormalities at the baseline electroencephalographic recording. Informed consent was obtained from all participants, and the study was approved by the Institutional Review Boards of Yuanpei University (YPU-IRB-1110426). The personal information of all participants, including age, gender, education, and history of illness, was recorded before starting the study. In this study, electroencephalography (EEG) was applied in order to record the brain waves of all participants. Heart rate and systolic and diastolic blood pressure were measured before and after drinking the tea infusion at each fixed time point (0, 7, 14, 21, 28 days).

### 2.4. Alpha Brain Wave Measurement

The α brain wave data were wirelessly collected from study subjects using frontal EEG devices in accordance with our previous study [15]. The NeuroSky^®^ MindWave Mobile headset (NeuroSky Inc., San Jose, CA, USA, henceforth MindWave) was used in our research, because it is one of the most popular, convenient, and affordable EEG devices at a low cost that is accessible for neuroscientists. The EEG frequency analysis was performed by means of a Fast Fourier Transform (FFT) algorithm, with a 2 s interval of EEG signals. A single-channel EEG procedure was designed to collect brain activity data from participants. With reference to the ear electrode, the EEG data were wirelessly acquired from FP1 at a 128 Hz sampling rate (FP1: front point 1, 1 cm above the midline of left eyebrow). Simplicity and efficiency were the main reasons for designing a single-channel EEG monitoring system. The electroencephalographic data were analyzed offline, and the following frequency bands were computed: delta (ranging from 0.1 to 3.0 Hz), theta (4.0 to 7.0 Hz), alpha (8.0 to 13.0 Hz), beta (14.0 to 30.0 Hz), and gamma (31.0 to 47.0 Hz). The electric activities of α brain waves (μV) in alpha frequency bands were measured at baseline and after drinking the tea infusion at different time points (0, 30 min, and 28 days). The values of α brain wave activity (between 0.01 μV and 1.00 μV) were converted to α brain wave scores (1–100).

### 2.5. QOL Questionnaire

Eight-item QOL questionnaires (scores range from 1 to 5) were also completed on day 28 after the daily consumption of the tea infusion. The QOL questionnaire was evaluated with a five-point Likert scale in eight dimensions: (1) Euphoria. (2) Feeling of relaxation. (3) Better sleep. (4) Fewer headaches. (5) Less muscle tension. (6) Less physical discomfort. (7) Improvement in concentration. (8) Helpful for subjective enjoyment of life.

### 2.6. Statistics

All data are expressed as mean ± standard deviation (SD) unless otherwise stated. Differences between the groups were calculated using one-way ANOVA with Dunnett’s multiple-comparisons test. All statistical analyses were performed using SPSS version 22.0 (SPSS, Inc., Chicago, IL, USA).

## 3. Results

### 3.1. The Comparison of pH Values between GO Tea and Various Tea Products

Various tea products obtained from local markets are described in Table 1. The pH values of various teas were also analyzed. The pH values of WP, HO, OB, TO, and GO teas were 5.84, 5.93, 4.95, 5.89, and 5.07, respectively. Notably, the GO and OB teas have a more acidic pH than other teas (*p* value < 0.05). According to prior research, an acidic condition could improve GABA production [12]. Our results are in accordance with a previous study reporting that an acidic condition (pH 4.5–5.5) could improve GABA production [12].

### 3.2. The Comparison of Phytochemicals between Various Tea Products

The contents of total phenolics, total catechins, and free amino acids in the various Taiwanese tea products are shown in Table 2. Most tea extracts contained high concentrations of total phenolic compounds and were rich in total catechins. However, GO tea appeared to have lower contents of polyphenols and catechins than other Taiwanese tea products. In the comparison of amino acid contents between various teas, we found the highest levels of free amino acids in GO tea (*p* < 0.05). HO tea also contains a high quantity of amino acids, which could demonstrate the difference between high-altitude tea and other ordinary tea products. An increase in the cultivation altitude reduces total polyphenol contents but increases the free amino acid concentration. This result is in accordance with a previous study on Lushan Mountain tea in China by Han et al. [16]. Collectively, the higher amino acid content in GO may arise from the cultivation of the tea plant at a high altitude and the use of more nitrogen fertilizer.

### 3.3. The Comparison of Compositions for Eight Catechins among Various Taiwanese Tea Extracts

In our prior study, the identification of eight major catechins was determined by HPLC according to retention times obtained from authentic standards run under identical conditions [13]. The major catechins in all tea leaves include (+)-catechin (C), gallocatechin (GC), (−)-epigallocatechin (EGC), (−)-epicatechin (EC), (−)-epicatechin gallate (ECG), and (−)-epigallocatechin gallate (EGCG), with EGCG and EGC being the most abundant in the tea products in this study (Table 3). The contents of the various catechins in the ordinary oolong teas were all higher than those in GO tea. Compared with the results of total polyphenols and total catechins in Table 2, there should be many unidentified polyphenolic compounds or catechins in GO tea.

### 3.4. The Anti-Hypertensive Effects of GO Tea Extracts

**Figure 1:**
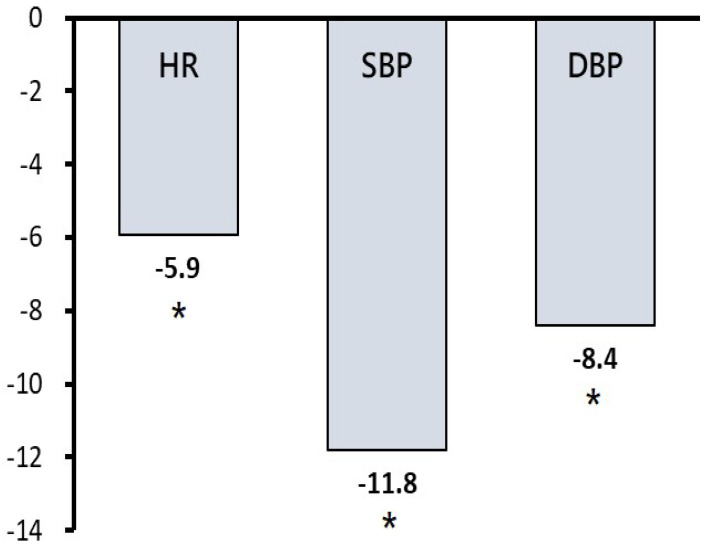
Effect of daily consumption of GO tea on the reduction in heart rate and blood pressure in the subgroup analysis for study participants with pre-hypertension after a period of 28 consecutive days. Abbreviations: DBP, diastolic blood pressure (mmHg); HR, heart rate (beats per minute); SBP, systolic blood pressure (mmHg). * Indicates a significant difference at the level of *p* < 0.05. Note: After a period of 28 consecutive days of GO tea consumption, study participants with pre-hypertension exhibited a significant decrease in heart rate and blood pressure.

GABA has been evidenced as a powerful bioactive compound with anti-hypertensive effects [17]. We conducted an interventional prospective cohort study design to investigate the anti-hypertensive effects of GO tea in the middle-aged population. Thirty-eight healthy volunteers (five males and thirty-three females) were included, with their characteristics described in Table 4. Table 4 demonstrates the baseline characteristics of the study subjects (*n* = 38) before consuming the GO infusion. The duration of the follow-up was twenty-eight days. The mean age of all study participants was 55.4 ± 10.4 years. Approximately 51.5% of the study participants had a history of pre-hypertension. Table 5 indicates that interval changes in the study subjects’ heart rate and systolic and diastolic blood pressure exhibited descending trends with the daily consumption of GO tea. Among all study participants, drinking GO oolong tea daily reduced systolic blood pressure on day 21 and diastolic blood pressure on day 28 (*p* < 0.05). To investigate further, data for subjects with pre-hypertension were extracted. Their baseline mean systolic blood pressure was 137.2 ± 15.6 mmHg. In this subgroup analysis (Figure 1), systolic and diastolic blood pressure were significantly reduced by 11.8 and 8.4 mmHg after 28 days of consumption of GO tea, respectively (*p* < 0.05). Moreover, their heart rates were also significantly reduced by 5.9 bpm after four weeks (*p* < 0.05). The current results confirm that the daily consumption of GO tea could effectively reduce heart rate and blood pressure in middle-aged adults with pre-hypertension.

### 3.5. The Relaxation Effects of GO Tea Extracts

**Figure 2:**
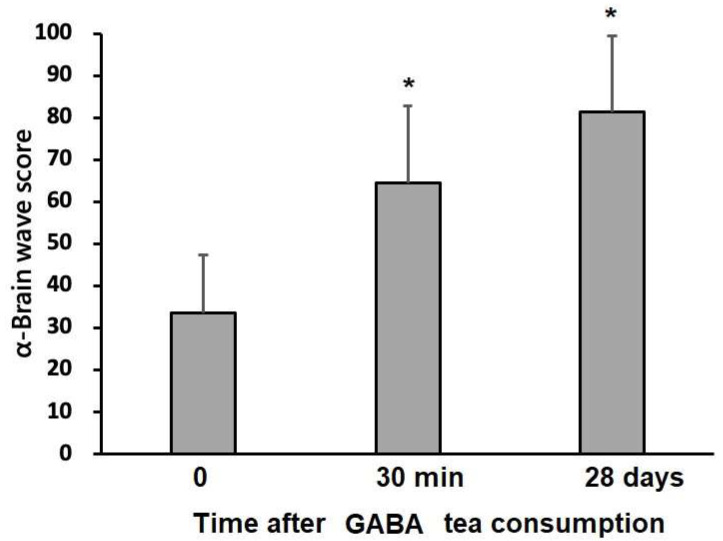
Effect of drinking GABA oolong tea on the α brain wave score. * Indicates a significant difference at the level of *p* < 0.05. Note: After 30 min and 28 consecutive days of GO tea consumption, study participants exhibited a significant increase in α brain wave score.

Throughout the study period, physical examinations revealed normal results in research participants, with no physical discomfort or abnormal laboratory data. Intriguingly, it is surprising that GO rapidly and effectively increased the alpha brain wave score after 30 min and four weeks, as shown in Figure 2 (*p* < 0.05). According to prior research, GO tea induces relaxation in a rapid and effective way [8]. In this study, GO tea contained abundant GABA and amino acids that could induce relaxation. It is worth noting that the α brain wave score related to relaxation was immediately increased by 1.8 times 30 min after drinking GO tea. Further, the α brain wave score was increased by 2.3 times after 28 days of continuous GO tea consumption. 

### 3.6. The QOL Improvement Associated with GO Tea Extracts

QOL questionnaires were further completed by all study subjects, including a pre-test and post-test, with a period of 28 days between tests. The eight dimensions are as follows: euphoria, relaxed feeling, better sleep, fewer headaches, less muscle tension, less physical discomfort, better concentration, and improved QOL (all *p* values < 0.05) (Table 6). A higher QOL score indicates higher satisfaction after 28 days of continuous GO tea consumption. We found that the daily consumption of GO for 28 consecutive days benefited the subjects’ stress relief, and all QOL indices were positively improved (Table 6).

## 4. Discussion

**Figure 3:**
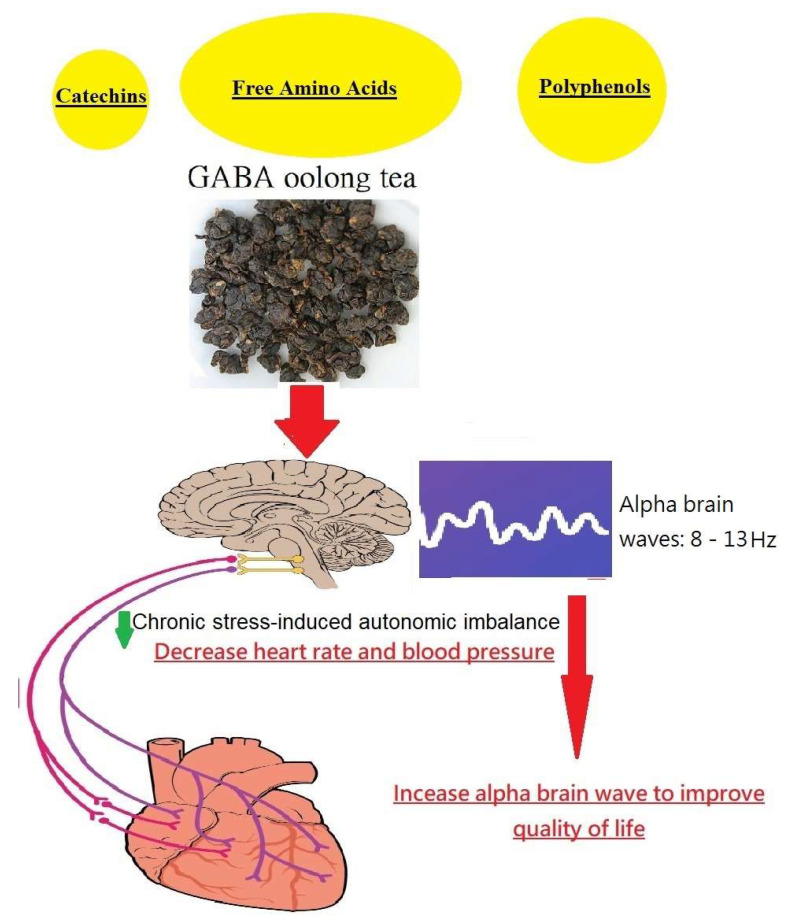
Graphical abstract of beneficial effects of GABA oolong tea on human health.

In this study, we developed a new manufacturing technology to produce a Taiwanese oolong tea product with high GABA content, which is termed GO tea. This study aimed to analyze the bioactive phytochemicals in GO tea via an HPLC colorimetric assay and compare the results with four famous Taiwanese tea products. In this interventional prospective cohort study design, encouraging results were obtained suggesting that GO tea can relieve mental stress, reduce blood pressure, and improve QOL in the middle-aged population. GO tea contains numerous bioactive constituents, including polyphenols, catechins, and amino acids (e.g., GABA), that synergistically act to improve stress-induced autonomic imbalance (increasing alpha brain waves and reducing blood pressure/heart rate), contributing to QOL and human health (Figure 3). For a better understanding of the underlying mechanism, several important findings in this work deserve further discussion.

### 4.1. Optimization of pH Condition in the Industrial Production of GO Teas

Lactic acid bacteria (LAB) are pivotal organisms in the fermentation of diverse food processing and function as GABA producers [12,18]. Myriads of raw foods and materials contain abundant glutamate that can be metabolized by LAB to enhance their tolerance to acidic conditions. As a result, such GABA-producing LAB have been utilized via isolation from a wide range of fermented foods, including cheese, kimchi, soybeans, and various fermented Asian fish products. To investigate further, we analyzed the pH values of various tea products. As expected, GO tea has a more acidic pH (5.07) than other teas (*p* value < 0.05). Our results are in accordance with a previous study indicating that an optimal pH condition (pH 4.5–5.5) could improve GABA production [12,18]. Our findings provide advantageous results for enhancing GABA production strategies and providing optimal conditions for food processing and industrial applications.

### 4.2. The Major Bioactive Phytochemicals in GO Teas: Pharmaceutical Roles of GABA

As shown in Table 2, GO tea appeared to contain lower levels of polyphenols and catechins than other Taiwanese tea products. In comparison, we found the highest levels of free amino acids in GO tea (*p* < 0.05). Although three famous Taiwanese tea products were found to contain trace GABA (below 1 mg/100 g) via the HPLC method, only GO contained 187 mg/100 g GABA in our prior analysis. GABA plays a critical role in the pathogenesis of hypertension, which is associated with targeting angiotensin-converting enzyme regulation [3]. Moreover, central GABA is able to decrease blood pressure and slow heart rate through the activation of GABA receptors [19,20]. When it comes to the diverse pharmaceutical properties of GABA, Ngo et al. comprehensively review them and illustrate GABA’s therapeutic effects on a myriad of diseases, particularly hypertension, diabetes, insomnia, anxiety, hypothyroidism, cancer, kidney injury, alcohol-related hepatotoxicity, infection, allergies, oxidative stress, inflammatory conditions, atherosclerotic disease, neurological disorders, etc. [5]. In light of the versatile beneficial effects of GABA, biotechnological techniques for high GABA production have been applied in the food industry. Although the preliminary results were encouraging in clinical applications, further testing and validation concerning the safety and efficacy of daily GABA consumption are necessary by conducting large-scale, randomized, placebo-controlled trials.

### 4.3. The Versatile Effects of GO Tea on Stress Relief and QOL Improvement

Information regarding changes in mental stress obtained from EEG signals remains scarce. Thus, we used the NeuroSky MindWave device to measure alpha brain waves in accordance with prior research [21,22]. Alpha electromagnetic waves ranging between 8 and 13 Hz in frequency and between 30 and 50 μV in amplitude are clearly enhanced in closed-eye periods and serve as an index of relaxation [21,22]. In our study protocol, the α brain wave score was measured before and after drinking the tea infusion at different time points (0, 30 min, and 28 days). The baseline α brain wave score was 35.0 ± 17.3. Intriguingly, it is surprising that GO rapidly increased the alpha brain wave score after 30 min (*p* < 0.05). As expected, GO tea induces relaxation in a rapid and effective way [8]. The above effect relies on the high contents of GABA and amino acids in GO tea, which can induce relaxation (Table 2). Notably, the α brain wave score related to relaxation was immediately increased by 1.8 times 30 min after drinking GO tea and 2.3 times after 28 days of continuous GO tea consumption. The potent relaxation effect resulted in a significant improvement in QOL (Table 6). A higher QOL score suggests higher satisfaction after 28 days of continuous GO tea consumption, including euphoria, relaxed feeling, better sleep, fewer headaches, less muscle tension, less physical discomfort, better concentration, and subjective enjoyment of life. We found that the daily consumption of GO for 28 consecutive days benefited the subjects’ feelings of relaxation, and all QOL indexes were positively improved (all *p* values < 0.05). To explore the above versatile properties more in depth, we reviewed the antioxidant effects of GABA on cytoprotection and the neuromuscular system. In a C2C12 myoblast cell model with oxidative damage, GABA therapy reduced reactive oxygen species (ROS) levels, reversed glutathione (GSH) depletion, and restored the activities of antioxidant enzymes such as catalase (CAT) and superoxide dismutase (SOD) so as to protect cellular homeostasis [23]. In the cerebral cortex and hippocampus of an acute epileptic rat model, GABA treatment diminished the malondialdehyde concentration and modulated the expression of SOD and glutathione peroxidase (GPx) [24]. Moreover, GABA not only reacted readily with malondialdehyde under physiological conditions but also trapped reactive intermediates during lipid peroxidation [25]. Emerging evidence has shown the protective effects of GABA against H_2_O_2_-induced ROS in pancreatic cells [26] and human umbilical vein endothelial cells [27], in which it was associated with reduced cell death, the inhibition of ROS production, and enhanced antioxidant defense systems. In the small intestine of rats with gamma-ray-induced oxidative injury, the cytoprotective effects of GABA were observed via decreases in malondialdehyde and advanced oxidation protein production, increased CAT and GPx activities, decreased mucosal damage and hemorrhage, and the promotion of the regeneration of intestinal cells [28]. In addition, brain oxidative damage in streptozotocin-treated rats was attenuated by GABA treatment [29]. Collectively, GABA ameliorated ROS and pro-inflammatory mediator production and suppressed inflammatory symptoms [3]. The potential mechanism and synergistic effects of GO tea contributing to human health are illustrated in Figure 3.

## 5. Conclusions

Our study confirmed that GO tea contained not only polyphenols but also significantly higher levels of amino acids, including GABA, contributing to whole-body relaxation and anti-hypertensive effects. Among study subjects with pre-hypertension, the daily consumption of GO tea could reduce heart rate and systolic and diastolic blood pressure. Not only alpha brain waves but also QOL was effectively improved. Evidence from our human study suggests that GO tea may potentially serve as a natural source for alternative therapy to lower mental-stress-related blood pressure elevation and improve QOL. The therapeutic effects of GO tea are versatile, and large-scale, placebo-controlled clinical trials are in urgent need.

## Figures and Tables

**Table 1:** Comparison of pH values between various tea products.

Tea	Abbreviation	Tea Cultivar	Place of Origin	pH
Wenshen Paochong tea	WP	Chin-Shin-Oolong	New Taipei	5.84 ^a^
High-mountain Oolong tea	HO	Chin-Shin-Oolong	Chiayi	5.93 ^a^
Oriental Beauty tea	OB	Chin-Shin-Dapan	Hsinchu	4.95 ^b^
Dongding Oolong tea	DO	Sijichun	Nantou	5.89 ^a^
GABA oolong tea	GO	Sijichun	Nantou	5.07 ^b^

Means not sharing a common letter were significantly different (*p* < 0.05). Note that the OB and GO teas have a more acidic pH than other teas (*p* < 0.05).

**Table 2:** Comparison of phytochemicals between various Taiwanese tea extracts.

	Total Phenolics mg GAE/g	Total Catechins mg GAE/g	Free Amino Acids mg TE/g
WP	404.4 ± 0.8 ^a^	164.9 ± 4.0 ^b^	73.4 ± 3.4 ^c^
HO	326.6 ± 1.8 ^c^	140.9 ± 1.8 ^c^	93.7 ± 4.6 ^b^
OB	328.6 ± 0.6 ^c^	147.2 ± 5.1 ^c^	68.4 ± 1.5d ^c,d^
DO	353.2 ± 2.7 ^b^	195.4 ± 1.2 ^a^	65.6 ± 2.3 ^d^
GO	263.0 ± 4.4 ^d^	91.0 ± 3.2 ^d^	99.0 ± 4.0 ^a^

Abbreviations: GAE, gallic acid equivalents; TE, theanine equivalents; WP, Wenshen Paochong tea; HO, High-mountain Oolong tea; OB, Oriental Beauty tea; DO, Dongding Oolong tea; GO, GABA oolong tea. Each value is expressed as mean ± standard deviation (*n* ≥ 3). Means not sharing a common letter were significantly different (*p* < 0.05). Note that free amino acid levels in WP, OB, and DO were significantly lower than those in GO tea. GO tea contains the highest levels of free amino acids.

**Table 3:** The comparison of compositions for eight catechins among various Taiwanese tea extracts.

	Content (mg/g Extract)				
	WP	HO	OB	DO	GO
Catechin	4.4 ± 0.1 ^b^	4.2 ± 0.1 ^b^	4.1 ± 0.0 ^b^	5.2 ± 0.1 ^a^	3.5 ± 0.1 ^c^
EC	10.6 ± 0.3 ^b^	11.2 ± 0.4 ^b^	9.7 ± 0.1 ^c^	12.9 ± 0.1 ^a^	6.2 ± 0.4 ^d^
ECG	9.5 ± 0.6 ^c^	8.2 ± 0.4 ^d^	11.7 ± 0.2 ^b^	17.2 ± 1.0 ^a^	5.8 ± 0.4 ^e^
EGC	66.1 ± 0.8 ^b^	74.6 ± 0.6 ^a^	9.0 ± 0.5 ^e^	52.3 ± 1.3 ^c^	12.1 ± 0.5 ^d^
EGCG	64.1 ± 0.6 ^b^	61.7 ± 1.6 ^b^	18.7 ± 0.2 ^c^	87.1 ± 0.9 ^a^	11.7 ± 0.8 ^d^
GA	1.3 ± 0.0 ^c^	0.8 ± 0.0 ^c^	10.8 ± 0.2 ^a^	0.9 ± 0.1 ^c^	7.6 ± 0.2 ^b^
GC	25.4 ± 0.6 ^a^	25.1 ± 1.8 ^a^	7.4 ± 0.7 ^c^	19.1 ± 0.7 ^b^	7.3 ± 0.4 ^c^
GCG	20.8 ± 1.9 ^a^	16.1 ± 1.45 ^b^	5.5 ± 0.2 ^c^	23.4 ± 2.7 ^a^	4.9 ± 0.1 ^c^

Tea abbreviations: WP, Wenshen Paochong tea; HO, High-mountain Oolong tea; OB, Oriental Beauty tea; DO, Dongding Oolong tea; GO, GABA oolong tea. Catechin abbreviations: EC, epicatechin; ECG, epicatechin gallate; EGC, epigallocatechin; EGCG, epigallocatechin gallate; GA, gallic acid; GC, gallocatechin; GCG, gallocatechin gallate. Means not sharing a common letter were significantly different (*p* < 0.05). Note that GA levels in WP, OB, and DO were significantly lower than those in GO tea.

**Table 4:** Baseline characteristics of the study subjects (*n* = 38).

Variables	Average
Age (years)	55.4 ± 10.4
Gender (female; *n* (%))	33 (86.8)
History of pre-hypertension (*n* (%))	17 (51.5)
Daily sleep time (hour)	7.00 ± 1.06
Heart rate (beats per minute)	74.9 ± 9.2
Systolic blood pressure (mmHg)	120.0 ± 20.0
Diastolic blood pressure (mmHg)	77.5 ± 12.4
Alpha brain wave score	35.0 ± 17.3
Education (Bachelor’s degree or higher)	21 (63.6)
Types of work (white-collar worker)	25 (75.8)

**Table 5:** Changes in heart rate and blood pressure after GABA oolong tea consumption for 28 days.

Variables	Before	GO Consumption			
	0	7	14	21	28
Heart rate (bpm)	74.87 ± 9.18	73.00 ± 9.89	72.30 ± 8.68	73.52 ± 9.05	72.76 ± 8.38
SBP (mmHg)	120.00 ± 20.04	117.33 ± 16.65	116.39 ± 12.69	114.52 ± 14.53 *	115.76 ± 12.89
DBP (mmHg)	77.5 ± 12.35	75.06 ± 9.02	74.55 ± 7.27	74.12 ± 8.49	73.52 ± 7.99 *

SBP, systolic blood pressure; DBP, diastolic blood pressure. GO, GABA Oolong tea. Each value is expressed as described in Table 3. * Indicates a significant difference at the level of *p* < 0.05.

**Table 6:** Changes in 8-item QOL questionnaire score before and after 28 consecutive days of GABA oolong tea consumption.

Item	Treatment	
	Before	After
Euphoria	3.4	4.3 *
Relaxation feelings	3.3	4.2 *
Better sleep	3.2	4.2 *
Fewer headaches	3.0	3.8 *
Less muscle tension	3.0	4.1 *
Less physical discomfort	3.1	4.0 *
Improvement in concentration	2.9	4.3 *
Helpful for subjective enjoyment of life	2.9	4.1 *

Each item is scored on a 5-point Likert scale. * Indicates a significant difference at the level of *p* < 0.05.

## Data Availability

The data used to support the findings of this study can be made available by the corresponding author upon request.
